# Considerations in the treatment of individuals with obesity and periodontitis

**DOI:** 10.1111/cob.70002

**Published:** 2025-03-26

**Authors:** Abigail S. Q. Cheong, Jean E. Suvan

**Affiliations:** ^1^ Faculty of Dentistry, Oral & Craniofacial Sciences King's College London London UK; ^2^ Oral Sciences, University of Glasgow Dental School, School of Medicine, Dentistry and Nursing, College of Medical, Veterinary and Life Sciences University of Glasgow Glasgow UK

**Keywords:** bariatric, diabetes, obesity, periodontal therapy, weight

## Abstract

Two common non‐communicable diseases, obesity and periodontitis, are responsible for and affected by systemic inflammation, sharing common risk factors and mechanistic pathways. Periodontitis is an irreversible immune‐mediated inflammatory disease of hard and soft tissue supporting teeth. If left untreated, periodontitis can lead to tooth loss, affecting food choices and healthy eating, therefore affecting overall health. Obesity is an independent predictive factor for worsened periodontal inflammation, increased onset, progression, severity, and recurrence of infection, as well as delayed wound healing. Thus, managing obesity and associated metabolic dysfunctions may improve periodontal therapy outcomes. The chronic inflammatory state of obesity impairs immune regulation exacerbating the inflammatory gingival tissue destruction of periodontitis, which can also systemically contribute to inflammatory mediators. Furthermore, bariatric surgeons and dietitians should educate patients with obesity regarding the risk of elevated caries, xerostomia, and periodontitis risk from acid reflux and frequent food intake. Non‐dental healthcare professionals should recognise periodontal disease signs to prompt dental referral when warranted. Asking patients about recent dental visits promotes patient involvement in cross‐discipline dialogue to enhance patient care coordination between medicine and dentistry. This article discusses the association between these two diseases, the challenges of achieving optimal periodontal treatment outcomes, and the clinical strategies to enhance holistic care. It also explores oral health considerations in dietary and surgical interventions in the treatment of obesity.

**Abbreviations**
BMI: body mass index
BS: bariatric surgery
DM: diabetes mellitus
NSPT: non‐surgical periodontal therapy
PMPR: professional mechanical plaque removal
T2DM: type 2 diabetes mellitus

## 1 INTRODUCTION

The global epidemic of obesity has wide‐ranging pathological impacts as the chronic inflammatory state of obesity exacerbates infection and impedes healing, therefore contributing toward adverse outcomes and complicating the treatment of periodontal disease, also referred to as periodontitis. Periodontitis is a bacterially induced, immune‐mediated inflammatory disease of the periodontium, the surrounding supporting structures of teeth (gingiva, periodontal ligament, cementum, and alveolar bone). Affecting 796 million people globally, this severe infection is largely influenced by several lifestyle factors such as excess sugar intake, tobacco use, and inadequate oral hygiene, common to many non‐communicable diseases.^1^ Public health initiatives targeting shared risk factors and promote good oral hygiene habits can significantly reduce the burden of oral diseases. Periodontitis also demonstrates bidirectional pathogenesis with other chronic inflammatory conditions such as diabetes mellitus, hypertension, and atherosclerosis, worsening systemic and periodontal outcomes.^2^, ^3^ Periodontal inflammation affects systemic health through bacterial pathways and inflammatory mediators, while these inflammatory conditions are known to impact periodontal health through altered immune response. Prioritising a balanced diet low in free sugars and high in fruits and vegetables, as well as advocating for water as the primary beverage, could yield substantial benefits. Additionally, emphasising the importance of brushing twice daily with fluoride toothpaste containing 1350–1500 ppm fluoride should be a key focus.^4^

Through a review of published research, this article aims to discuss obesity as a modifying factor of clinical periodontitis treatment outcomes and discuss implications for managing patients with obesity and periodontitis across dental and healthcare settings. Clarifying how obesity influences periodontal disease severity and treatment success could provide further guidance for clinical decision making and inform multi‐disciplinary, patient‐centred care approaches for this population. Oral health considerations for those treating obesity include current oral health status and close monitoring for signs of periodontal disease progression.

## 2 PATHOGENESIS AND AETIOLOGY OF PERIODONTITIS

**FIGURE 1:**
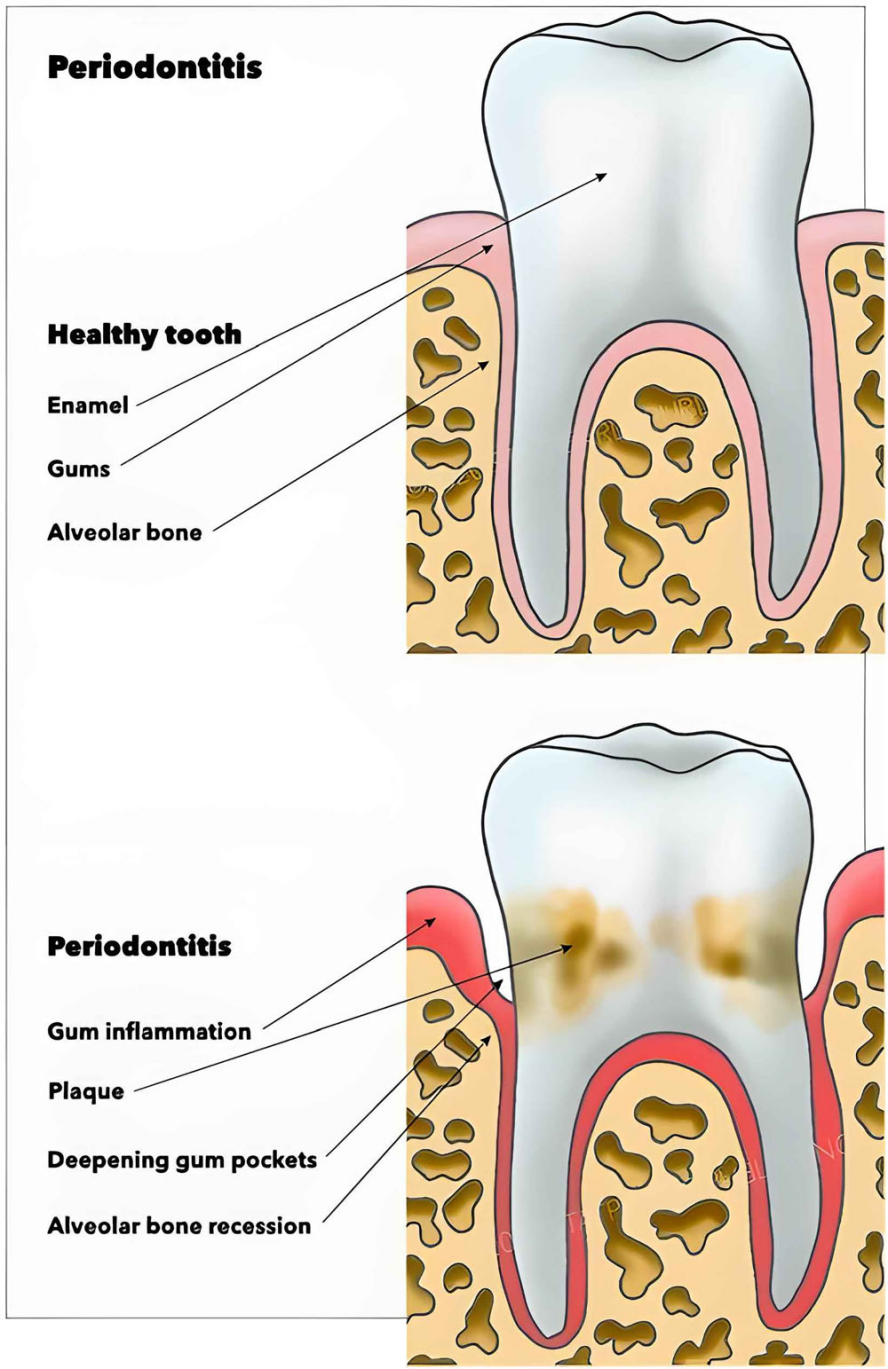
Diagrammatic image of healthy periodontium and periodontitis.^5^

The disease progression reflects tissue damage from inflammation and infection of periodontal tissue structures, including the gingiva, periodontal ligament, cementum, and alveolar bone. Managing periodontitis is imperative to stop disease progression. Signs of gingival health include firmness and pink colouration of gingiva tissue. Indicators of periodontitis include halitosis, swollen or red gingiva, alveolar bone loss, loose teeth, tooth loss, bleeding from brushing, pus discharge between the teeth and gums, and jaw pain or tenderness (Figure 1).^5^

Virulent periodontal pathogens, local environment, and host susceptibility alter frequency and severity of the disease presentation, which is also largely influenced by the individuals' risk factor status. Risk factors for periodontitis fall into two categories: modifiable factors such as smoking and poor oral hygiene, while non‐modifiable factors include age and genetic susceptibility.^6^ Periodontitis intrinsically heightens host immunoinflammatory dynamics against infection; however, in patients with obesity and co‐occurring diabetes mellitus, the host response exhibits additional immune dysfunction and heightened inflammatory activity that precipitates accelerated periodontal attachment loss and tissue destruction.^7^ Periodontitis has a multi‐factorial aetiology, with plaque being the primary aetiological factor. Local factors such as calculus, overhanging restorations, and carious lesions promote the accumulation of dental plaque, which can harden into calculus, causing gingivitis, the initial stage of periodontitis.^8^ Gingivitis is reversible with good oral hygiene habits such as brushing twice a day with a manual or electric toothbrush and interdental brushes, and visits to a dental professional for removal of calculus (hardened plaque). However, if not managed, gingivitis can progress to periodontitis, an irreversible disease that requires continuous professional management and good oral hygiene habits at home to prevent the continuous, irreversible damage to the periodontal ligaments, resorption of alveolar bone, and subsequent tooth loss.^6^

## 3 OBESITY

Similar to periodontitis, obesity can be categorised as a chronic inflammatory condition, with adipose tissue dysfunction eliciting systemic inflammation through aberrant adipokine signalling, macrophage infiltration, and dysregulation of cytokine production. Obesity is one of the most prevalent non‐communicable chronic diseases and can be defined as having too much body mass, with a body mass index (BMI) of ≥30 in adults.^9^ Obesity is associated with an increase in several inflammatory markers, leading to chronic low‐grade inflammation.^10^ Although easily measured, BMI has limitations including body builders and athletes, who have more muscle and may have higher BMI scores even though their fat levels are low. In spite of the downfalls, BMI remains a valuable tool to indicate obesity‐related health risks such as hypertension, atherosclerosis, specific cancers, diabetes mellitus, cardiovascular diseases, and musculoskeletal disorders, which result in a dramatically decreased quality of life and life expectancy.^3^ In accordance with BMI classifications, individuals exhibiting progressive obesity face an elevated susceptibility to the onset of type 2 diabetes mellitus (T2DM), hypertension, and cardiovascular disease.

## 4 AETIOLOGY OF OBESITY

The aetiology of obesity is multifactorial, involving complex interactions between genetic, biological, behavioural, and environmental factors.^11^ Obesity primarily results from long‐term energy imbalance between consumed calories and expended calories. Sedentary lifestyles with minimal physical activity contribute to weight gain by minimising energy output, while excessive intake continues.^11^ Storing excess caloric consumption as fat for extreme conditions, such as starvation, is an ingenious biological mechanism acquired during evolution. However, in modern obesogenic environments with easy access to high‐calorie food and low physical activity levels, this previously adaptive trait can become maladaptive, contributing to overweight and obesity.

Specific gene variants and hormone imbalances such as leptin, ghrelin, insulin, and cortisol can alter appetite regulation, metabolism, and fat storage.^12^, ^13^ Diets abundant in processed, calorie‐dense, and high‐fat foods contribute to fat accumulation. Meanwhile, excess intake of sugary foods and beverages disrupts appetite control. Ethnic groups differ in body fat percentage and metabolic risks at the same BMI level. For example, people of Asian descent tend to have more body fat and higher metabolic risk compared with Caucasians.^14^ Socioeconomic status and health behaviours are examples of factors that, if improved, can contribute to reduced obesity levels and healthier individuals.

## 5 LINK BETWEEN OBESITY AND PERIODONTITIS

**FIGURE 2:**
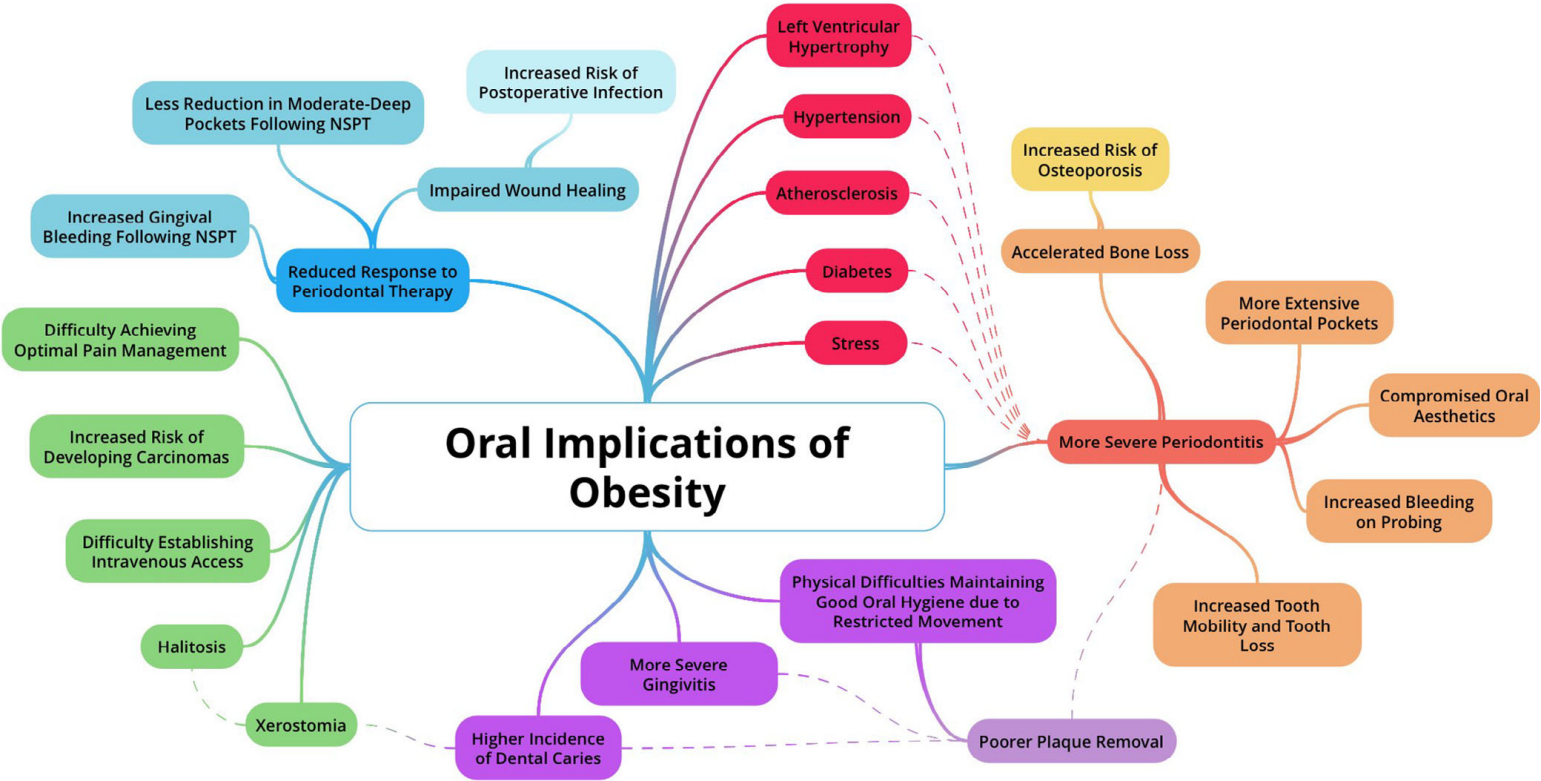
Oral implications on obesity.

Contrary to previous assumptions, extensive research has proven adipose tissue to be responsible for metabolism and energy conversion as well as a fat storage medium.^11^ Although the combination of mechanisms in which obesity predisposes individuals to periodontitis remains unknown, epidemiological investigations indicate obesity as a salient risk factor, predisposing afflicted individuals to amplified vulnerability to developing severe periodontitis (Figure 2).^15^ This supports older analysis carried out on 13 665 adults, published in 2003, that found obesity among young adults to be associated with a higher prevalence of periodontal disease. While being underweight, BMI <18.5 kg/m^2^, is associated with a lower prevalence of periodontitis, independent of traditional risk factors (including age, gender, and cigarette smoking).^16^ Furthermore, the pathophysiological interplay between obesity and oral health encompasses impaired mastication and disruptions in performing oral hygiene due to excess adipose tissue physically obstructing access.

Development of insulin resistance as a result of a chronic inflammatory state and oxidative stress could be implicated in the association between obesity and severe progression of periodontitis.^15^, ^17^ A comparative study compared oxidative stress levels in patients with and without obesity, presenting with various periodontal conditions. Detailed analysis found oxidative stress higher in individuals with obesity compared with individuals without obesity in all groups: those with chronic periodontitis, gingivitis, and periodontal health.^18^ This suggests oxidative stress, inflammation from obesity, and periodontal disease has a synergistic effect. In addition to adequate physical activity, strategies to reduce oxidative stress focus on a plant‐based diet, avoiding oxidative triggers such as smoking, and managing underlying conditions such as obesity.^19^ Adopting lifestyle changes can help patients restore their redox balance for optimal health.

Adipose tissue is an interactive organ in the endocrine system, related to the signalling cascade via the secretion of cytokines and chemokines, promoting inflammatory signalling.^20^ Therefore, individuals with obesity may present with a higher incidence of infection.^21^, ^22^ Due to the persistent impact of obesity‐related factors on oral health, a combination of obesity‐related factors such as chronic inflammation and impaired immune function can lead to a higher risk of periodontitis recurrence, delayed wound healing, and amplified severity of periodontitis.^23^, ^24^ Proposed pathological mechanisms for the association of obesity and periodontitis also implicate deregulation of lipid metabolism. The low‐grade chronic inflammation and immune cell activation observed in obesity are seen to disrupt homeostatic functions in adipose tissue, leading to immune cell infiltration and activation.^23^ Leptin is an adipose‐derived hormone that stimulates pro‐inflammatory responses.^25^ The obesogenic environment can increase circulating leptin levels, which accelerates metabolic dysfunctions, resulting in a hyperphagic status and adipose tissue expansion. Hyperleptinaemia is common in most individuals with obesity and reflects increased adiposity and leptin resistance, causing generalised sympathetic activation, stimulation of vascular inflammation, and oxidative stress.^26^ These factors are associated with complications such as hypertension, atherosclerosis, and left ventricular hypertrophy.^27^ Research suggests that maintaining specific levels of circulating leptin is crucial to prevent disturbances in nutrient consumption.^12^

Obesity increases the risk of developing T2DM, which has been identified as a major risk factor that can accelerate the progression and severity of periodontitis.^7^, ^28^ A systematic review on non‐experimental, epidemiological studies on the impact of diabetes mellitus on periodontal disease outcomes found evidence that periodontitis is worsened with uncontrolled glycaemic levels and the development of T2DM.^7^ Clinical presentations among those with both T2DM and periodontitis include impaired wound healing, more extensive pocketing, and accelerated bone loss.^7^, ^24^ Controlling obesity reduces the risk of T2DM and consequently limits the secondary outcome of more severe periodontitis. Appropriate management of underlying T2DM in patients with this comorbidity is critical to avoiding aggravated periodontal inflammation.

## 6 IMPLICATIONS OF OBESITY ON PERIODONTITIS TREATMENT

Obesity has been proven to be an independent predictor of a worse response to periodontal therapy, indicated by a prospective study assessing the periodontal status of moderate to severe chronic periodontitis at follow‐up periods of 2 and 6 months.^29^ The link between periodontitis and obesity is likely to be bidirectional, with inflammatory molecules shared between the two conditions.^30^ Periodontal patients are recommended treatment options that correspond to the stage of their periodontal disease. The accumulation of *Porphyromonas gingivalis* on teeth initiates the formation of plaque, a thin film that adheres to tooth enamel. Without diligent removal through brushing and flossing, plaque progresses into hardened calculus.^31^ Early intervention with good oral hygiene and routine dental visits for professional mechanical plaque removal (PMPR) can arrest periodontitis or prevent the progression of gingivitis to periodontitis. However, once the disease manifests, symptoms are irreversible and require continuous monitoring.

In 2022, a retrospective analysis of 3443 adult dental records was assessed and categorised based on BMI and periodontal severity. The study controlled for initial periodontal disease, age, gender, tobacco, and history of diabetes mellitus. A logistic regression model was used** to analyse the association between BMI and periodontal treatment intensity score, based on treatment type, number of teeth treated, and number of visits that were evaluated with multivariable negative binomial regression. The study concluded that patients with obesity had a 40% higher treatment intensity score compared with normal‐weight patients, suggesting that patients with obesity may require more intensive periodontal treatment independent of initial disease severity.^32^

### 6.1 Non‐surgical periodontal therapy

Non‐surgical periodontal therapy (NSPT) routinely starts with the removal of hard and soft accumulations from the tooth surface, which is sometimes sufficient as the sole treatment.^33^ The efficacy of NSPT in the management of periodontitis is well established, and clinical trials have shown a reduction of inflammation, pocket depth reduction, and clinical attachment gains following therapy.^33^ Maintenance therapy involves the mechanical removal of bacterial biofilm and deposits via mechanical (power and manual) instrumentation, creating a local environment favourable to better periodontal health. Providing oral hygiene advice, followed by an integration of good oral hygiene habits routinely carried out by the patient, aims to reduce gingival inflammation and reduce the progression of periodontitis.

Individuals with obesity and periodontitis can significantly benefit from NSPT. However, clinical improvements are less prominent in individuals with obesity with periodontitis compared with individuals without obesity with similar periodontal status due to chronic inflammation.^34^ This is further supported by the results of a study comparing periodontal treatment outcomes of 18 patients with and 18 patients without obesity. Plaque index, bleeding on probing, probing depth, and clinical attachment level were measured, with overall improved periodontal parameters improved for both test groups over 6 months. However, patients with obesity had less reduction in moderate‐deep pockets (>5 mm) than patients in the normal‐weight BMI category. The study concluded obesity negatively influenced outcomes for deeper pockets, associating obesity with poorer responses to periodontal treatment, especially for deeper pocket depths.^35^ A more recent clinical study published in 2023 similarly concluded that patients with obesity demonstrated poorer decreases in periodontal disease progression compared with patients without obesity following NSPT. This was evidenced by less improvement in the abundance of adipocytokines and oxidative stress markers among the patients with obesity.^36^

T2DM, a disease commonly associated with obesity, poses significant risks in the management of periodontal disease. T2DM has been associated with increased severity of periodontitis, including more extensive pocket formation and alveolar bone loss.^7^ These factors can reduce the effectiveness of periodontal treatment. Nevertheless, NSPT remains the recommended initial treatment approach even for patients with T2DM and periodontitis. However, patients with T2DM may require more frequent periodontal maintenance and monitoring due to impaired immune functioning and wound healing. Treatment outcomes in this population are likely to be less predictable and not as effective. Close clinical supervision and individually tailored treatment are crucial for optimal management of periodontitis in patients with T2DM.^32^

It should also be noted that evidence has shown that the treatment of periodontitis in patients with diabetes can improve HbA1c levels.^37^ Furthermore, nonsurgical periodontal therapy has been shown to lower biomarkers of systemic inflammation, including hsCRP, TNF‐α, IL‐1β, and improve endothelial function.^38^ These systemic benefits of periodontal therapy have also been shown to occur in patients with obesity.^39^

In addition to poorer plaque removal and increased bleeding after NSPT, clinical studies have shown that obesity correlates with reduced bone mass. This bone loss can result in varying degrees of osteoporosis and resorption of the alveolar bone surrounding the teeth, similar to periodontitis presentations.^40^ This could be due to physical difficulties accessing all areas for optimal home care. Patients with obesity may have difficulties maintaining good oral hygiene due to restricted movement. Periodontal therapy combined with weight loss programmes leads to better clinical improvements in attachment loss, pocket depths, and inflammation compared with periodontal treatment alone. Simultaneously addressing obesity and periodontal health produces optimal outcomes. However, periodontal treatment success relies upon regular, diligent patient adherence/engagement to maintain self‐performed oral hygiene.^41^

Controlling obesity is key to better periodontal outcomes. Other non‐surgical treatment options include antibiotics, antimicrobial agents, and laser therapy. However, obesity can affect the pharmacokinetics of antibiotics, potentially leading to suboptimal drug concentrations. This could compromise antibacterial effects and contribute to antibiotic resistance.^42^

### 6.2 Surgical periodontal therapy

Periodontal surgical treatment options include periodontal access flap surgery, regenerative or bone augmentation procedures, all of which are secondary treatment options to non‐surgical therapy. Potential periodontal treatment outcomes may be affected by obesity‐related risk factors such as impaired wound healing, increased risk of infections, difficulty achieving optimal pain management, and technical challenges.^28^ Delayed wound healing increases the risk of postoperative infection.^24^ The increased adipose tissue in the neck and throat regions can obstruct the airway, causing discomfort for patients with obesity in the supine position. Addressing these challenges requires clinicians to have a thorough understanding of the implications of obesity on airway management and to adopt strategies to treat patients holistically, ensuring the safety and well‐being of patients with obesity throughout dental care.

Difficulty or inability to establish intravenous access has also been documented.^43^ Administering local anaesthetic to patients with obesity presents challenges due to excess soft tissue obscuring anatomical landmarks needed for procedures such as inferior alveolar nerve blocks. The abundance of soft tissue can cause difficulties with palpating sites for injection placement.^44^ Furthermore, dosages for many drugs are weight‐based. Standard doses of emergency drugs, based on pharmacokinetics obtained in people of average weight, may not be appropriate for patients with obesity.^42^ Considering these factors, patients with obesity are at a potentially greater risk of medical emergencies occurring. Obesity adds complexity to periodontal surgery due to impaired visualisation and access from enlarged soft tissues, necessitating the need to account for pathological adiposity and associated anatomical alterations in optimising surgical planning for patients with obesity. Increased thickness of oral soft tissues coupled with visual obstruction during flap procedures may impede surgical accuracy and complexity, increasing the risk of complications.^45^

## 7 EFFECT OF WEIGHT‐LOSS ON ORAL STATUS

Approaches to treat established obesity include dietary therapy, increasing physical activity, cognitive behavioural therapy, pharmacotherapy, and surgery.^46^ Bariatric surgery (BS), a surgical intervention to treat obesity, has the initial goal of reducing the patient's stomach size to reduce storage food absorption capacity, resulting in weight loss and reduction and/or cure of correlated disease.^47^ Oral health consequences of BS include increased risk of dental caries, wear, hypersensitivity, salivary flow alterations, and periodontal disease due to chronic regurgitation and increased food intake at shorter intervals. The frequent occurrence of oral pH imbalance throughout the day, due to an increased number of daily meals necessary for a balanced diet creates an acidic environment, is associated with a higher incidence of caries, gingivitis, and worsening periodontal disease.^48^ The reduction in salivary flow decreases salivary buffering, which leads to an increased susceptibility to the development of dental caries and halitosis.^49^ Therefore, bariatric surgeons can prevent or halt the rate of progression of periodontal disease by promoting healthy nutrition and adequate physical activity preoperatively and postoperatively.^16^ Success in obesity prevention and therefore periodontitis will require management through thorough characterisation of the intersections between genetics, physiology, environmental, and social behaviours that influence regulatory mechanisms.^50^ Before BS, patients should be referred to dental professionals for an oral health check‐up to gauge if their oral health is being managed. This allows for early intervention to prevent exacerbation of oral health diseases.

## 8 MANAGEMENT OF PATIENTS WITH OBESITY IN THE DENTAL PRACTICE

Barriers to providing treatment to patients with obesity include the physical environment and medical complications. Patients with periodontitis often necessitate complex interventions and interdisciplinary management from a team of periodontal specialists and surgeons. This is particularly crucial as the severity of the disease is often heightened, coupled with compromised healing capabilities, which can elevate the risk of infection recurrence, also supported by a recent prospective study carried out in 2020.^51^ Given these complexities, dental practitioners must vigilantly monitor patients with obesity throughout the entirety of their periodontal treatment and post‐treatment. Preoperative care should focus on monitoring the patient's blood pressure and medications, as obesity is often linked to additional health issues such as hypertension.^52^ During treatment, BMI in combination with other information from the patient's medical and social history should be used to determine an appropriate treatment plan. This includes asking targeted medical history questions to determine the level of disease and airway management risks, as patients with obesity are also likely to have additional health problems such as hypertension. Post‐treatment, it is imperative to monitor wound recovery to effectively mitigate disease advancement and ensure optimal patient outcomes.^22^ A high BMI, leading to increased subcutaneous deposits around the buccal region and an inflamed tongue, makes treatment access challenging.^44^ Clinicians may have to alter operator positioning when carrying out treatment to increase proximity to the patient's oral cavity.

Dental professionals can/should evaluate patients for signs and symptoms of obesity‐related diseases. Dental caries, periodontitis, and xerostomia are examples of oral health problems linked to obesity.^53^ An older survey of periodontists (*n* = 103) and general dentists (*n* = 105) found a concerning gap in the communication between dentists and physicians to coordinate care for patients with T2DM. The survey revealed that periodontists engage in more frequent risk assessments and management practices for patients with diabetes mellitus or a smoking history compared with general practitioners.^54^ Enhanced communication and collaboration among members of the healthcare team, including dietitians and general practitioners, is required to enable integrated management of comorbidities with periodontitis, such as T2DM. Dentists can refer patients for weight loss support and discuss weight reduction interventions such as diet therapy so patients can have improved control over their periodontal inflammation.^55^

Healthcare providers face unique barriers in caring for patients with obesity, including physical accessibility issues that require forethought and accommodation. Severe obesity may limit mobility, preventing housebound patients from accessing dental clinics.^44^ Even ambulatory patients may encounter obstacles from parking facilities to the dental suite itself, including narrow doorways, small corridors, and cramped restrooms. Practices should identify and mitigate these barriers by offering assistance and ensuring accessible entry routes and comfortable waiting areas with armless, high‐weight‐capacity seating. The combination of limited mobility and restricted intra‐oral access, due to increased subcutaneous deposits, necessitates extended appointment durations to optimise procedural efficiency. Operating considerations also warrant attention, such as blood pressure cuffs and weighing scales. Modern dental chairs have a maximum lifting weight of ~140 kg (23 stones).^44^ Solutions include treating patients on an operating table, trolley, or custom‐made bariatric dental chairs. Addressing access limitations will allow providers to better care for patients with obesity.^56^

## 9 PATIENT PERCEPTIONS OF WEIGHT ASSESSMENT

A profound body of evidence indicates that obesity acts as a modifying factor for periodontitis, with implications such as poorer responses to periodontal therapy and increased risk of carcinomas. Effective communication between practitioners and patients should be instilled to deliver related health messages such as promoting the maintenance of a healthy weight. The given evidence raises questions as to whether recording patient weight assessments should be implemented in routine dental care to emphasise how obesity‐fuelled chronic inflammation impacts overall periodontal and oral health. Directly inquiring about patient weight opens dialogues about monitoring weight, diet, and exercise habits. Alternatively, self‐reported BMI could be considered, although this may introduce limitations involving intentional or unintentional underreporting. A 2019 cross‐sectional multicentre study involving 213 adults (aged 20–85) across four private dental clinics in London and Hampshire assessed patient acceptance of healthy weight promotion from dental professionals. While most patients supported dentists promoting healthy weights, those with obesity displayed significantly higher sensitivity about this issue.^57^ Overall awareness regarding the risks of periodontal disease, cancer, and oral health issues linked to obesity was low among survey participants. This points to a need for healthcare providers, as also recommended by the WHO, to provide more holistic health guidance.^58^

## 10 IMPLICATIONS FOR CLINICAL PRACTICE

This article reviews the periodontal treatment outcomes in patients with obesity. Most of the evidence discussed has been published within the last 5 years, supported by seminal studies from the preceding three decades, providing a comprehensive understanding of primary research in this domain. Previously published research has suggested a positive association between obesity and the prevalence, progression, and severity of periodontitis, independent of age, gender, ethnicity, and plaque levels among study participants. However, due to individual predisposing patient risk factors, varying severities of periodontitis presentations, and adverse lifestyles, establishing a direct causal relationship between obesity and periodontitis has been challenging, as control groups are difficult to establish. Factors such as dental appointment frequency and patient compliance with oral hygiene, which are crucial in periodontitis outcomes and management, are not easily quantified and controlled. Nevertheless, studies have reported an adverse effect of obesity on periodontal treatment outcomes.^7^, ^15^, ^16^, ^17^, ^18^, ^21^, ^22^, ^23^, ^24^, ^29^, ^36^ A large survey (*n* = 13 665) estimating the association between increased body weight and periodontal disease found obesity as a potential risk factor for periodontal disease, especially among younger individuals with obesity.^16^

### 10.1 Dental professionals

Dental professionals and other healthcare professionals are well‐positioned to provide dietary guidance such as limiting calorie‐dense foods and drinks that impact obesity and periodontal inflammation. Nutritional counselling aligns with oral health promotion. Advising patients to moderate sugary food and drink intake allows dental teams to address obesity prevalence and nutrition behaviours that exacerbate periodontitis. As periodontitis has been associated with overall systemic health, dietary counselling presents an opportunity to improve patients' oral health and general well‐being. In the management of T2DM, maintaining good glycaemic control is imperative not only to reduce cardiovascular risks but also other complications, which have an adverse bearing on the health of an individual and affect the quality of life.^59^ Changes in medication related to T2DM therapy, use of anti‐inflammatory or antibiotics and alteration of lifestyle, including exercise and diet should be recorded and monitored. Each patient's treatment plan will be unique, considering individual patient characteristics, systemic health, and lifestyle factors.

### 10.2 Other healthcare providers

A basic strategy physicians can employ to enhance coordination of oral‐systemic care is inquiring when patients last visited a dentist. By stimulating patient recall and dialogue about oral health, physicians encourage patients to take a more active role in managing oral‐systemic links with their overall health. Enquiring may also reveal previously undetected dental issues, spur patient motivation to schedule upcoming dental appointments, and provide diagnostic clues through patient reports of specific oral systems associated with obesity. Additionally, physicians should strive to expand their knowledge regarding the symptoms of periodontal diseases. Equipping physicians to recognise complaint patterns, clinical presentations, and risk factors indicative of periodontal pathology enables them to then refer patients to dental or periodontal specialists when examination and treatment are warranted for suspected disease.^55^ Particularly for periodontal disease, regular assessment of oral health is critical for prompt detection of infection and tissue damage. Through comprehensive clinical exams and detailed reviews of systemic biomarkers and risk factors like glycaemic control and smoking, clinicians can profile progression. Patient‐centred care should include lifestyle and behavioural risks contributing to obesity, periodontitis, and associated inflammatory comorbidities. Collaborative, multidisciplinary medical and dental treatment plans can then align to not just resolve acute symptoms but foster overall health.

## 11 IMPLICATIONS FOR FURTHER RESEARCH

To better understand the relationship between obesity and periodontitis, more prospective, randomised controlled studies with large sample sizes are required. These studies should be representative of a range of periodontal disease severity and should categorise participants' medical histories, with adjustments for potential confounders such as T2DM. T2DM is intricately linked with both obesity and periodontal inflammation, further complicating the analysis of the obesity–periodontitis relationship. Controlling for T2DM and other potential confounders is critical to isolate the independent effects of obesity on periodontal disease progression and treatment outcomes. Additionally, factors such as appointment frequency and patient compliance with oral hygiene, which are known to significantly influence periodontitis outcomes, should be carefully monitored and quantified in these studies. Such research will inform clinical decision‐making and the development of targeted, multi‐disciplinary treatment strategies for patients with obesity and periodontitis. Furthermore, further research surrounding the oral implications of weight loss interventions including dietary and BS is warranted.

## 12 CONCLUSION

The current body of evidence supports the role of obesity as a modifier influencing periodontitis severity and treatment outcomes. Specifically, obesity exacerbates clinical presentations of periodontal inflammation: increased periodontal pocketing, increased onset, progression, and severity of periodontitis, as well as delayed wound healing and increased recurrence of infection and tissue destruction through chronic systemic inflammation. Thus, managing obesity and associated metabolic dysfunctions may lessen the incidence and progression of periodontitis in affected individuals.

Healthcare professionals across medicine, dentistry, and allied disciplines share an essential role in continually evaluating patients with obesity. Implementation of population health strategies promoting healthy nutrition, regular physical activity, and methods of managing associated chronic inflammatory diseases may further mitigate periodontal disease onset and progression. Large‐scale interventions focused on obesity control, avoidance of modifiable risk factors of periodontitis such as smoking, and managing confounders such as T2DM could further improve periodontal treatment outcomes. While periodontal therapy remains effective in obese patients, managing underlying comorbidities such as T2DM is imperative, as bidirectional interactions with periodontitis exacerbate outcomes for both diseases. A multifaceted public health approach targeting inflammation at oral and systemic levels through lifestyle changes, education, improved access, and coordinated care may overcome determinants fuelling global rising rates of periodontal pathology.

## AUTHOR CONTRIBUTIONS

Abigail S. Q. Cheong carried out literature searches, the generation of original figures, and the primary writing of the manuscript under the guidance of Jeanie E. Suvan. Both Abigail S. Q. Cheong and Jeanie E. Suvan contributed to the design and content of the manuscript. Abigail S. Q. Cheong and Jeanie E. Suvan both contributed to the content, editing, and final approval of the submitted and published versions.

## CONFLICT OF INTEREST STATEMENT

The authors have no known conflicts of interest for this publication.

## Data Availability

The data that support the findings of this study are available on request from the corresponding author. The data are not publicly available due to privacy or ethical restrictions.
